# The prevalence, antimicrobial sensitivity, and factors associated with camel mastitis in Isiolo County, Kenya

**DOI:** 10.14202/vetworld.2022.2962-2970

**Published:** 2022-12-28

**Authors:** Willy Edwin Mwangi, George Karuoya Gitau, Davis Ikiror, Peter Kimeli, Moses Irungu Gakuru, Douglas Machuchu, Wallace Kingori

**Affiliations:** 1 Department of Clinical Studies, Faculty of Veterinary Medicine, University of Nairobi, P. O. Box 29053-00625 Nairobi, Kenya; 2 Vétérinaires Sans Frontières Suisse, Muthangari Road, off Gitanga Road, P. O. Box 25656-00603, Nairobi, Kenya; 3 County Ministry of Agriculture, Livestock and Fisheries, Isiolo County Government, P. O. Box 36-60300, Isiolo, Kenya

**Keywords:** camel, drug sensitivity, mastitis, risk factors

## Abstract

**Background and Aim:**

The one-humped camels (*Camelus dromedarius*) adapt very well to arid and semi-arid (ASALs) environments and continue to thrive and produce milk even during severe droughts when cattle, sheep, and goats experience high mortalities. These attributes make the dromedaries very vital for the survival of pastoralists, especially in the ASALs of Africa. Mastitis is among the most important diseases of camels and can cause significant economic losses, thereby endangering the livelihoods of pastoralists. This study aimed to estimate the prevalence, risk factors, and antimicrobial sensitivity of mastitis-causing pathogens in lactating camels in Isiolo County, Kenya.

**Materials and Methods:**

This was a cross-sectional study conducted in July and August 2021. A questionnaire was administered to the camel keepers to capture data on herd-level factors. Lactating camels were then examined for any visible signs of clinical mastitis and as well as to capture data on other animal-level factors such as age, weight, and body condition score. A composite milk sample was collected aseptically from randomly selected camels in a herd. The samples were initially tested for somatic cell counts (SCC) using Somatos mini^®^ automatic cell counter. Culture and sensitivity testing was then carried out on any milk sample that had SCC ≥ 200,000 cells/mL. The descriptive analysis was initially used to summarize the continuous and categorical farm and camel factors, while mixed regression models were used to explore the association between/across mastitis and the farm as well as camel-level factors.

**Results:**

A total of 210 lactating camels from 23 herds were selected and sampled in this study. The average age of camel keepers was 48.3 ± 16.3 years and they were involved in rearing camels for a mean of about 14.3 ± 8.6 years. The total number of camels in a herd ranged from 10 to 287, with the mean total herd milk production being 34.5 ± 24.9 L/day. The mean daily milk production per camel was 2.8 ± 1.7 L while the range for days in milk was between 21 and 787 days. The average age of camel first calving and the inter-calving interval was 56.3 ± 9.9 and 27.7 ± 9.9 months, respectively. The median parity of the dam was three and the body condition score was two. About 39.7% (83/210) of the sampled camels had clinical mastitis during the current lactation. The overall prevalence of mastitis (SCC ≥ 200,000 cells/mL) in camels was 17.6%. The main pathogens isolated were *Streptococcus* and *Staphylococcus* bacteria. *Streptococcus* isolates were mainly sensitive to ampicillin and resistant to cefaclor. All 18 *Staphylococcus* isolates were sensitive to tetracycline, while 12/18 isolates were resistant to ampicillin. The factors that were significantly associated with mastitis were the age of the respondent (p = 0.038), the number of years involved in camel rearing (p = 0.024), presence of clinical mastitis since calving (p = 0.039), body condition score (p = 0.040), and age of the dam at the time of examination (p = 0.072).

**Conclusion:**

Results from this study revealed that mastitis is an important condition among camels in the pastoral communities of Isiolo County, Kenya. Thus, pastoralists should continue to be aware of and trained on mastitis occurrence and its control in the pastoral ecosystem to reduce potential economic losses.

## Introduction

The one-humped camels (*Camelus dromedarius*) adapt very well to arid and semi-arid (ASALs) environments and continue to thrive even during severe droughts when cattle, sheep and goats experience high mortalities. Camels are largely browsers, a tenet that assists them in accessing feed from trees and shrubs during droughts when pastures are scarce. Although camels are raised in harsh environments, they are able to produce milk consistently and in adequate quantity [1]. Camel milk contributes significantly to pastoralists’ diet and income generation [2]. These attributes make the dromedaries very vital for the survival of pastoralists, especially in ASALs part of Africa [3]. In Kenya, the camel is the most important dairy animal in the ASALs and produces approximately 340 million liters of milk annually [4].

Mastitis is among the most important diseases of camels and has a worldwide distribution [5]. The disease can be either clinical or subclinical [6–8] and is associated with significant economic losses. These losses are attributed to the cost of treatment, reduced milk production, compromised quality of milk and public health concerns due to the widespread use of antimicrobials [9–11]. Sub-clinical mastitis is the most prevalent yet the less diagnosed form of the disease in camel dairy herds [12]. Consequently, sub-clinical mastitis is the form that is attributed to the highest economic losses [3, 12]. Clinical mastitis can also occur but is easily recognized and managed by most camel keepers [13].

Past studies have shown that udder infections in lactating camels are widespread. In Africa, mastitis in camels has been reported in Egypt [6, 14]; Somalia [8]; Ethiopia [7, 15]; and Kenya [5, 16, 17]. The reported prevalence of camel mastitis under pastoralism in Kenya ranges from 46 to 93.3% [5, 16, 17]. Different bacteria have been isolated from mastitic mammary glands in camels, either in the form of pure or mixed infection. *Streptococcus agalactiae* and *Staphylococcus aureus* are the frequent etiology of mastitis in camel herds in Africa [5, 13, 17]. There is a paucity of data on antimicrobial sensitivity patterns of pathogens isolated from camel milk. However, a previous study found widespread tetracycline resistance among *S. agalactiae* isolates from camel milk in northern Kenya [17]. In addition, *S. aureus-*resistant genes have been identified in camel milk in Kenya and Somalia [18]. The diagnosis of mastitis is achieved either indirectly through leukocyte counts or changes in milk composition or directly through the identification of the causative agent [13]. In camels, factors such as age, parity, stage of lactation, season, and teat-tying to prevent the calf from suckling have been associated with subclinical mastitis [12, 13, 17].

Reducing the number of new mastitis infections is the major goal of any mastitis prevention program and can be achieved by optimizing milking procedures and post-milking teat disinfection [19]. These practices can reduce the number of shedders in the herd, separate the shedders from the uninfected camels, and optimize the immune function of the animal, which are key components of decreasing new infections [19]. Eliminating existing infections reduces the exposure of susceptible quarters and may be obtained by treatment during lactation or at dry-off, or by culling of the infected animals [20]. Again, the separation of the infected animals from the susceptible group may also be an effective method to limit the exposure of susceptible animals and reduce the risk of new infections [19].

This study aimed to estimate the prevalence and determine the risk factors of mastitis in camels raised by pastoralists in Isiolo County, Kenya, and further establish the main causative pathogens and their antimicrobial sensitivity to camels.

## Materials and Methods

### Ethical approval

The study was approved by the Faculty of Veterinary Medicine, University of Nairobi Biosafety, Animal Use and Ethical Committee (Approval no. FVMBAUEC/2021/295).

### Study period and location

This was a cross-sectional study conducted in July and August 2021 in Isiolo County, which is a typical ASAL area located in the north eastern region of Kenya. Isiolo borders Marsabit County to the North, Wajir County to the East, Garissa and Tana River Counties to the southeast, Meru County to the South, Laikipia County to the southwest and Samburu County to the West. The County has 10 Wards distributed in three sub-counties: Isiolo township, Merti, and Garbatulla. The administrative headquarter is in Isiolo town. The County covers an area of 25,700 km^2^ with a total human population of about 268,000 individuals [21]. Geographically, Isiolo County lies between 0° 21’ South and 37° 35’ East. The altitude is between 200 and 1,100 m above sea level with an average elevation of 769 m. The rainfall pattern is bimodal, unpredictable and erratic in distribution. Long rains occur from late March to May while short rains occur from November to December. The annual average rainfall range is between 350 and 600 mm while the mean annual temperature is between 24°C and 30°C. Livestock keeping is the major economic activity in the County and is a key source of livelihood. The main livestock species found in Isiolo County include camel, goats, sheep, cattle, donkeys, and poultry. Although traditionally, cattle have been the main animal species raised in the county, there has been a gradual change toward camel keeping. This shift has been occasioned by resilience of the camel to droughts, limited pasture and water resources and new economic opportunities especially increased demand for camel milk and meat. The 2019 Kenya national census estimated the total camel population in Isiolo County to be around 148,859 [21]. Although Isiolo County is cosmopolitan, the camel-keeping households are mainly ethnic Somalis, Borana, Garre, Samburu, and Turkana. Isiolo County is also home to several wildlife conservancies with a significant population of free-roaming wildlife, resulting in human-domestic animals-wildlife interaction.

### Selection of households

Four administrative wards and households where data were collected were purposively selected based on the availability of camel herds, security situation, proximity to Isiolo town, and accessibility. From each of the four administrative wards, 5–6 households were targeted for the detailed study to make a total convenient sample of 23 households.

### Sample size calculation

To calculate the sample size, a prevalence of 26% was used at 95% level of confidence and 5% error of estimation. The 26% prevalence of camel mastitis was previously reported in a similar study Seligsohn *et al*. [17]. The sample size was computed using the formula described by Dohoo *et al*. [22], as shown below:


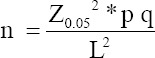


(L = 0.05 margin of error, p = 0.26 prevalence of camel subclinical mastitis previously reported in a similar study, q = 1-p = 0.74 and Z_0.05_ is the normal deviation from the mean in Z distribution = 1.96). The calculated sample was at least 296 lactating camels.

### Household data collection

Data on farm management practices were collected using a structured questionnaire which was administered to the head or a representative who was familiar with the household and the camel herd. The local administrative officers assisted in identifying and locating the households and camel herds within a selected area. The interviews were conducted by a pre-trained enumerator who was proficient in English, Gabbra, Somali, and Oromo. The questionnaires included household demographics such as family size, gender of the household head, level of education, and herd structure such as the number of camels, age categories among others. Other data collected were individual camel data (age, parity, days in milk, previous history of mastitis, ongoing treatments, and general condition) and herd data (herd management and risk factors for mastitis). The interviews were conducted during or after the sampling of milk from lactating camels.

### Selection of lactating camels

From each herd, a maximum of 20 lactating camels were selected for sampling. In herds with fewer than 20 lactating females, all lactating females were sampled. In herds with more than 20 lactating females, every second camel to be milked was sampled until a maximum target of 20 camels was met. To reduce sampling bias, herders were asked whether a milking order existed. In a few cases, the herders used a milking order based on the age of the calves, with calves divided into different age groups. In such circumstances, equal numbers of camels were randomly selected and sampled from each calf age group.

### Milk sample collection

Before sampling, animal owners and/or herders were informed about the purpose of the study and the sampling procedures, and their oral consent to take part was obtained. Initially, the udder was palpated and checked for blind teats and signs of clinical mastitis, such as lesions, swelling, heat, pain, induration, and deviating color. The presence of blind teats or signs of clinical mastitis were recorded on site. In addition, the selected lactating camels were examined and information appertaining to body condition score, body weight, the color of mucus membrane, status of superficial lymph nodes, intermandibular space, abdomen, and hair coat were noted and recorded.

Milk samples were collected for laboratory analysis to determine somatic cell count, bacterial isolates, and antimicrobial sensitivity. Milk sampling took place during the first-morning milking. Milk let-down was then initiated by allowing the calf to suckle. About 5 mL of milk was collected aseptically from each quarter and pooled directly into two sterile collecting tubes. The tubes were then labeled appropriately and immediately kept in cool boxes packed with frozen gels awaiting transportation to Isiolo County veterinary laboratory. Once in the laboratory, one tube of 10 mL was removed from the cool box and allowed to attain room temperature. Thereafter, the somatic cell count from these milk samples was determined using an automatic cell counter (Somatos mini^®^, Sibagropribor Ltd, Krasnoobsk, Russia) while following the manufacturers’ instruction. Any milk sample with a somatic cell count (SCC) reading of >200,000 cells/mL was considered to be mastitic. Milk samples from the remaining tube of 10 mL were frozen at −20°C awaiting transportation to the bacteriology laboratory at the Department of Clinical Studies, Faculty of Veterinary Medicine, University of Nairobi, where bacterial isolation and antimicrobial sensitivity tests were performed. Samples were cultured 5–7 days after collection.

### Bacterial culture and sensitivity

Bacterial culture and sensitivity were carried out as recommended by Patel [23]. Briefly, frozen milk samples were left to stand on a bench and thawed to room temperature (29°C). A loopful (Approximately 10 μL) of milk sample was aseptically streaked on blood agar supplemented with 5% defibrinated sheep blood (Department of Clinical Studies, Faculty of Veterinary Medicine, University of Nairobi, Nairobi-Kenya) and another 10 μL on MacConkey agar plate (HiMedia Laboratories LLC, Kennett Square, Pennsylvania, USA). The two plates were then incubated aerobically at 37°C for 24 h. Samples with more than 200,000 SCC/mL but displaying negative growth on the primary culture were subjected to extend plating. Pure culture was further obtained by sub-culturing part of typical and well isolated colony on a corresponding medium and incubated further aerobically at 37°C for 24 h. Identification of bacteria isolates was then made on the basis of colony morphology (size and color); hemolytic reactions on blood agar; lactose fermentation on MacConkey agar; Gram staining reactions (Gram-positive vs. Gram-negative and cocci versus rods); and biochemical test. For biochemical tests, a catalase reaction was performed as previously described by Karen [24] to differentiate *Streptococcus* and *Staphylococcus* species.

The antimicrobial used for the sensitivity test was Ampicillin, Tetracycline, Streptomycin, Kanamycin, Gentamycin, Norfloxacin, and Cefaclor (Table-1). (All antibiotic disk were from HiMedia Laboratories LLC). The antimicrobial sensitivity of *Streptococcus* and *Coccobacillus* isolates was tested against ampicillin, cefaclor, and tetracycline, while those of *Staphylococcus* isolates were tested against ampicillin, gentamicin, cefaclor, kanamycin, tetracycline, and norfloxacin. The antimicrobial susceptibility testing was done by the agar disk diffusion method as described by the Clinical and Laboratory Standards Institute [23]. In brief, a 0.5 McFarland standardized suspension of the bacteria was prepared in 0.85% sterile normal saline solution. A sterile cotton swab was dipped into the standardized suspension of bacteria and then uniformly streaked over the entire surface of the Mueller-Hinton agar (HiMedia Laboratories LLC). Notably, the organism coccobacillus failed to produce visible growth on Mueller Hinton agar and was therefore streaked on blood agar supplemented with 5% defibrinated sheep blood. After streaking, paper disks impregnated with a fixed concentration of antibiotics were placed on the agar surface and incubated in an inverted position at 37°C for 24 h. After 24 h, clear zones of inhibition were produced by the bacterial growth and diffusion of the antibiotics. The zones of inhibition were measured in millimeters using a caliper and interpreted as susceptible, intermediate, and resistant.

**Table-1:** The concentrations of antimicrobials used for sensitivity testing.

Antimicrobial	Disk concentration
Ampicillin	25 μg
Tetracycline	25 μg
Streptomycin	10 μg
Kanamycin	30 μg
Gentamycin	10 μg
Norfloxacin	10 μg
Cefaclor	30 μg

### Data management and analysis

Data were initially entered into Microsoft Excel 2010 (Microsoft, Washington, USA), where they were cleaned and sorted and, imported to Stata 15.1 software (https://www.stata.com/stata15/) for statistical analyses. Proportions were determined for categorical variables, while mean, median, standard deviation, and range were calculated for continuous variables. The apparent prevalence of mastitis in lactating camels was determined as a proportion of positive camel samples from the total samples collected.

A multilevel mixed-effects logistic regression analysis was performed to identify risk factors associated with mastitis (SCC ≥ 200,000 cells/mL) in camels. The SCC counts of <200,000 were considered negative (coded 0), while those ≥200,000 were considered positive (coded 1). In the first step, univariable regression analysis for all the predictor variables was fitted into separate models to determine their unconditional associations with the presence of mastitis. In the second step, multivariable logistic regression analysis was fitted for all the univariable associations with p ≤ 0.3. Initially, correlations between predictor variables were evaluated using pair-wise correlation. The final model was fitted manually through backward stepwise removal of variables with least statistical significance while retaining variables with p ≤ 0.05. Plausible biological interactions between significant explanatory variables in the final model were also tested [22]. The area under the curve of the receiver operating characteristic was used to evaluate the overall model performance.

## Results

### General household demographics and animal characteristics

Demographics, farm, and animal characteristics are shown in Tables-2 and 3. In summary, 210 lactating camels from 23 herds were selected and sampled in this study. Most of the respondents were male (91.3%) with an average age of 48.3 ± 16.3 years and were involved in rearing camels for a mean of about 14.3 ± 8.6 years. Over 90% of the respondents were married, and only 4.4% indicated having formal education, mainly primary level. Households had a family size of between 4 and 12 individuals, with land ownership (residential land) being predominantly private. The total number of camels in a herd ranged from 10 to 287, with the other common livestock species raised being goats and sheep. The mean total camel milk production in a herd was about 34.5 ± 24.9 L/day with the average age of camel first calving and inter-calving interval being about 56.3 ± 9.9 and 27.7 ± 9.9 months, respectively.

**Table-2:** Household demographics and farm characteristics for the camel herds in Isiolo County, July and August 2021.

Continuous variables					

Variable	Number	Mean	SD	Median	Range
Age of the respondent (years)	23	48.3	16.3	53.0	4.0–72.0
The number of people in the primary household	23	8.9	2.7	10.0	4.0–12.0
Number of years involved in camel rearing	23	14.3	8.6	13.0	1.5–30.0
Total number of camels in the herd	23	91.4	83.3	55.0	10.0–287.0
Total number of cattle owned	23	4.5	15.0	0	0–54.0
Total number of goats owned	23	50.1	72.8	0	0–285
Total number of sheep owned	23	26.2	45.5	0	0–148
Total number of donkeys owned	23	1.8	1.8	2	0–7
Distance covered to access pasture (Km)	23	7.2	3.7	7.0	3.0–15.0
Distance covered to access water (Km)	23	4.7	2.7	4.0	2.0–11.0
Total milk yield/camel herd/day	23	34.5	24.9	20.0	5.0–80.0
Average age of the camel at first calving (months)	23	56.3	9.9	60	36–72
Average calving interval of the camel (months)	23	27.7	9.9	24	12–48
Average weaning age of the camel calves (months)	23	13.3	3.6	12	12–24

**Categorical variables**					

**Variable**	**Category**	**Number**	**Proportion (%)**		

Gender of the respondent	Male	21	91.3		
	Female	2	8.7		
Highest level of education attained by the respondent	None	22	95.7		
	Primary	1	4.4		
Marital status of the respondent	Married	21	91.3		
	Single	2	8.7		
Position of the respondent in the family	Husband	19	82.6		
	Wife	3	13.0		
	Child	1	4.4		
Land ownership status (residential)	Private	15	65.2		
	Communal	8	34.8		
Animal number per sub-county area	Isiolo	141	67.1		
	Garbatulla	69	32.9		

**Table-3:** Characteristics of lactating camels in Isiolo County, July and August 2021.

Continuous variables (n = 210)				

Variable	Mean	SD	Median	Range
Somatic cell count	222331.4	355704.3	90000	90000–1500000
Age of the dam at the time of examination (years)	10.1	3.8	9.75	4–30
Parity of the dam at the time of examination	3.4	2.1	3	1–12
Body condition score	2.1	0.5	2	1–5
Live body weight (Kgs)	306.4	28.3	306	178–386
Current daily milk yield from the dam (L)	2.8	1.7	2.5	0.5–21
Days in milk	223.0	106.0	209	21–787

**Categorical variables (n = 210)**				

**Variable**	**Category**	**Number**		**Proportion (%)**

Presence of clinical mastitis since calving	No	127		60.5
	Yes	83		39.5

The average age and weight of lactating camels sampled in this study were 10.1 ± 3.8 years and 306.4 ± 28.3 Kg, respectively. The mean daily milk production per camel was 2.8 ± 1.7 L while the range for days in milk was between 21 and 787 days. The median parity of the dam was three and the score for body condition was two. About 39.5% (83/210) of the sampled camels had clinical mastitis during the current lactation.

### Prevalence, culture, and antimicrobial sensitivity

**Figure-1:**
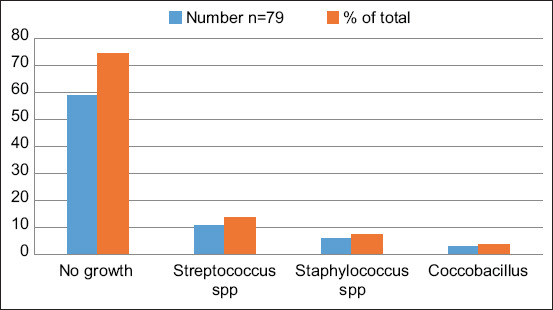
Bacterial isolate from 79 milk samples collected from camels in Isiolo County, Kenya during July and August 2021.

The overall prevalence of subclinical mastitis (SCC ≥ 200,000 cells/mL) in camels in Isiolo County was 17.6% (37/210). Of the 79 samples that were submitted for culture and isolation, 74.7% (59/79) showed no growth. *Streptococcus* spp. was isolated in 13.9% (11/79) of the samples, *Staphylococcus* spp. was isolated in 7.6% (6/79), and *Coccobacillus* was isolated in 3.8% (3/79) of the milk samples (Figure-1). Antimicrobial sensitivity results are summarized in Table-4. Briefly, *Streptococcus* isolates were mainly sensitive to ampicillin and resistant to cefaclor. All *Coccobacillus* isolates were resistant to tetracycline and only 4/6 isolates were sensitive to both ampicillin and cefaclor. All 18 *Staphylococcus* isolates were found to be sensitive to tetracycline with the highest resistance observed in ampicillin.

**Table-4:** The antimicrobial sensitivity patterns against the three bacteria species isolated from camel milk.

Bacterial spp.	Ampicillin	Cefaclor	Tetracycline	Gentamicin	Kanamycin	Norfloxacin
*Streptococcus* n = 11						
S	81.8% (9)*	9.1% (1)	54.5% (6)			
I		9.1% (1)	36.4% (4)			
R	18.2% (2)	81.8% (9)^#^	9.1% (1)			
*Coccobacillus* n = 6						
S	66.7% (4)*	66.7% (4)*				
I	33.3% (2)					
R		33.3% (2)	100% (6)^#^			
*Staphylococcus* n = 18						
S	33.3% (6)	83.3% (15)	100% (18)*	83.3% (15)	83.3% (15)	83.3% (15)
I						
R	66.7% (12)^#^	16.7% (3)		16.7% (3)	16.7% (3)	16.7% (3)

S=Sensitive, I=Intermediate, R=Resistant.

# Indicate the most resistant antibiotic in a particular bacterial isolate.

* Indicate the most sensitive antibiotic in a particular bacterial isolate

### Factors associated with mastitis in camels

The following factors were found to have a univariable association (p ≤ 0.3) with mastitis in camels. Age of the respondents, number of years involved in camel rearing, parity of the dam, body condition score, and presence of clinical mastitis since calving (Table-5).

**Table-5:** Univariable associations of predictor variables with the occurrence of subclinical mastitis in lactating camels in Isiolo County, July and August 2021.

Variable	Type	p-value
Highest level of education attained by the respondent	Categorical	0.735
Number of years involved in camel rearing	Continuous	0.267*
Total number of camels in the herd	Continuous	0.965
Total milk yield per day	Continuous	0.976
Age of the dam at time of examination (years)	Continuous	0.722
Parity of the dam at time of examination	Categorical	0.183*
Body condition score	Categorical	0.225*
Live body weight		0.543
Current daily milk yield from the dam	Continuous	0.602
Days in milk	Continuous	0.846
Animal number per sub-county area	Categorical	0.656
Presence of clinical mastitis since calving	Categorical	0.052*

* Variable with p values ≤ 0.30

In the final mastitis infection model, five variables: age of the respondents, number of years involved in rearing camels, presence of clinical mastitis since calving, age, and body condition score of the camel were found to be significantly associated (p ≤ 0.05) with mastitis (Table-6). A unit increase in the age of the respondents reduced the odds of camels having mastitis by 3% while a unit increase in the number of years involved in camel rearing increased the odds of camels having mastitis by 5%. The odds of a camel having mastitis increased by 155% when the body condition score was more than 2 and by 105% when the age of the dam at the time of sampling was >10.5 years. The odds of having mastitis were 2.3 times higher when milk was sampled from a camel that had suffered clinical mastitis in the current lactation.

**Table-6:** Final multilevel mixed-effects logistic regression model of subclinical mastitis in 210 lactating camels in Isiolo County, July and August 2021.

Variable	Category	OR	95% CI	p-value
Age of the respondent (years)	Continuous	0.97	0.94, 0.99	0.038
Number of years involved in camel rearing	Continuous	1.05	1.01, 1.10	0.024
Presence of clinical mastitis since calving	No	Baseline		
	Yes	2.26	1.04, 4.88	0.039
Body condition score	≤2	Baseline		
	>2	2.55	1.04, 6.23	0.040
Age of the dam at time of examination (years)	≤10.5	Baseline		
	>10.5	2.05	0.94, 4.47	0.072

## Discussion

The 17.6% prevalence of subclinical camel mastitis observed in this study was lower than that reported previously in sub-Saharan Africa [5–7, 13, 16, 17]. Further, the findings that *Streptococcus* species of bacteria were the common causative agent of camel mastitis agree well with the reports from other studies in Kenya [5, 17]. There is a paucity of data and information related to the epidemiology of *Streptococcus* in camels but previous reports suggest that the bacteria could be associated with chronic mastitis [17]. In dairy cattle, *S. agalactiae* is a contagious udder pathogen that is transmitted within the herd by contaminated milking equipment and compromised milk hygiene. This can be considered a key pathway that *Streptococcus* species could have been transferred within the camel herds. This argument is founded on our observation and the fact that most of the respondents in this study neither washed the milking containers nor their hands in between milking camels and where they did, the quality of water was questionable. Other factors that could be associated with *S. agalactiae* mastitis in camels include udder/teat lesions such as camel pox [13, 25]; increased age and parity of the camel [6, 12]; and late stage of lactation [25, 26]. The isolation of coccobacillus from sampled camels with mastitis was unexpected and requires further investigation.

To the best of our knowledge, this is the first study to report the association between mastitis and the age of farmers/herdsmen and the number of years involved in keeping camels. Three possible explanations could elucidate the observed association between mastitis and the age of farmers/herdsman. One is attributed to the experience and accumulated knowledge gained through training and from extension services by the diverse programs/projects. The knowledge and experience would mean that the camel keepers adopt the best management practices, including those that aim to reduce the risk of mastitis in a herd [27]. Second, the farmers who were advanced in age had bigger herd sizes (correlation coefficients 0.28, p < 0.0001) and their camel herds produced more milk (correlation coefficients 0.27, p = 0.0001). High productivity meant that the farmers had a more substantial financial ability to invest in the herd’s best practices and biosecurity measures. Finally, the advanced age of the farmer could potentially mean that the person has a wealth of knowledge on diagnosis and treatment of mastitis in their camels and can purchase antibiotics to treat any symptomatic animal in the herd. As such, sampling from such a herd has potential bias as most animals would have recovered from any clinical disease due to the use of antibiotics.

The observation that mastitis increases with the years involved in keeping camels could be attributed to two factors. First, farmers with many years of experience keeping camels had bigger herds (correlation coefficients 0.35, p < 0.0001). Large camel herds demand intense management practices such as high hygiene standards during milking, which may be challenging to achieve in pastoral areas (due to scarcity of potable water), thus resulting in a higher infection rate. Second, there is always a tendency by the older farmer to maintain old management practices, such as teat tying that have been shown to predispose camels to mastitis [17]. This is unlike younger keepers, who are more open to change and ready to implement protective practices.

The observed association between higher body condition scores and higher prevalence of mastitis is in agreement with what has been reported in dairy cattle, where it was demonstrated that a higher risk of clinical mastitis occurred in cattle with high body condition scores and from the third parity [28]. This is attributed to the fact that animals that have optimal body condition score are healthy and are higher milk producers [29]. Since milking in the community where this study was conducted is usually done once per day, and then it means that most of these high milk-producing camels are more often left with milk in the udder. Milk is a good medium of bacterial growth and thus predisposes the udder to mastitis when it is not completely stripped. Other factors could be attributed to the increased risk of ketosis in over-conditioned animals [30]. A positive association between ketosis and clinical mastitis in cows has been previously documented [31], and is believed to be a result of reduced generation of chemoattractants to recruit leukocytes to the infected quarter, and an attenuated leukocyte response in the presence of ketone bodies [32].

The presence of mastitis in the current lactation was a risk factor of mastitis in this study and is in agreement with a previous report [17]. In this study, mastitis was assessed by measuring the somatic cell count and considered any sample with a reading of equal to or above 200,000 as having mastitis as previously reported in dairy cattle [33]. When the udder is affected by mastitis, leukocytes are deployed by the body to fight the infection [34] and thus, higher mastitis diagnosis in such animals. However, it should be noted that among the samples that were submitted for bacterial culture, 74.7% yielded no culture despite some having high somatic cell counts. The absence of bacteria on culturing could be attributed to chronic aseptic mastitis, to non-shedding of bacteria at the time of sampling, to intramammary infection in remission [35], or to fluctuating SCC due to physiological and environmental factors [36]. Furthermore, the recovery of bacteria from milk samples could have been affected by the freezing process before culturing, as previously reported [37].

## Limitations of the study

The sample size used in the study was 210 camels as opposed to the estimated sample size of 296 camels. However, the sample size of 210 camels was still considered adequate for the statistical estimations, given that the pastoral production system in the area is fairly homogeneous. In addition, the migratory nature of the camels in the study area may have also denied access to all the potential camels for the study.

## Conclusion

The results from this study revealed that the age of the respondents was negatively associated with the occurrence of mastitis, while the number of years involved in rearing camels, presence of clinical mastitis since calving, body condition score, and age of the dam were positively associated with mastitis in lactating camels. The isolation of coccobacillus bacteria causing mastitis was exceptional. The study recommends that the extension packages used for training camel keepers on mastitis control highlight the importance of both management and camel-level factors, emphasizing the five factors above. Further, it is a recommendation that detailed studies be conducted to estimate the prevalence and risk factors of coccobacillus mastitis.

## Authors’ Contributions

WEM: Designed the study, collected data, and drafted the manuscript. GKG and DI: Designed the study and proofread the manuscript. PK: Collected and analyzed data MIG and WK: Collected data and proofread the manuscript. DM: Designed the study and collected data. All authors have read and approved the final manuscript.
